# A Systematic Review of Semaglutide’s Influence on Cognitive Function in Preclinical Animal Models and Cell-Line Studies

**DOI:** 10.3390/ijms25094972

**Published:** 2024-05-02

**Authors:** Raluca Oana Tipa, Daniela-Gabriela Balan, Mihai-Teodor Georgescu, Luciana Angela Ignat, Ileana Adela Vacaroiu, Dragos Eugen Georgescu, Laura Raducu, Doina Andrada Mihai, Liviu-Vasile Chiperi, Andra-Elena Balcangiu-Stroescu

**Affiliations:** 1 Department of Psychiatry, “Carol Davila” University of Medicine and Pharmacy, 020021 Bucharest, Romania; 2 “Prof. Dr. Alexandru Obregia” Clinical Psychiatric Hospital, 041914 Bucharest, Romania; 3 Discipline of Physiology, Faculty of Dentistry, “Carol Davila” University of Medicine and Pharmacy, 050474 Bucharest, Romaniaandra.balcangiu@umfcd.ro (A.-E.B.-S.); 4 Discipline of Oncology, Faculty of Medicine, “Carol Davila” University of Medicine and Pharmacy, 020021 Bucharest, Romania; 5 Doctoral School, “George Emil Palade” University of Medicine, Pharmacy, Science and Technology of Targu Mures, 540142 Targu Mures, Romania; 6 Discipline of Nephrology, Faculty of Medicine, “Carol Davila” University of Medicine and Pharmacy, 020021 Bucharest, Romania; 7 Discipline of General Surgery, Faculty of Medicine, “Carol Davila” University of Medicine and Pharmacy, 020021 Bucharest, Romania; dragos-eugen.georgescu@umfcd.ro; 8 Discipline of Plastic and Reconstructive Surgery, Faculty of Medicine, “Carol Davila” University of Medicine and Pharmacy, 020021 Bucharest, Romania; 9 Discipline of Diabetes, Nutrition, and Metabolic Diseases, “Carol Davila” University of Medicine and Pharmacy, 020021 Bucharest, Romania

**Keywords:** semaglutide, cognitive, potential, effect, review

## Abstract

Since we aim to test new options to find medication for cognitive disorders, we have begun to assess the effect of semaglutide and to conduct a review gathering studies that have attempted this purpose. This systematic review focuses on the cognitive effects of semaglutide, a glucagon-like peptide 1 receptor agonist (GLP-1 RA), in the context of neurological and cognitive impairment. Semaglutide, a synthetic GLP-1 analog, showcased neuroprotective effects beyond metabolic regulation. It mitigated apoptosis and improved cognitive dysfunction in cerebrovascular disease, suggesting broader implications for neurological well-being. Also, studies highlighted GLP-1 RAs’ positive impact on olfactory function in obese individuals with type 2 diabetes, on neurodegenerative disorders, multiple sclerosis, and endotoxemia. In order to analyze current studies that assess the impact of semaglutide on cognitive function, a literature search was conducted up to February 2024 on two online databases, MEDLINE (via PubMed) and Web of Science Core Collection, as well as various websites. Fifteen studies on mice populations and two studies on cell lines were included, analyzed, and assessed with bias-specific tools. The neuroprotective and anti-apoptotic properties of GLP-1 and its analogs were emphasized, with animal models and cell line studies demonstrating enhanced cognitive function. While promising, limitations include fewer studies, highlighting the need for extensive research, particularly in the human population. Even though this medication seems promising, there are significant limitations, one of which is the lack of studies on human subjects. Therefore, this review aims to gather current evidence.

## 1. Introduction

**Figure 1:**
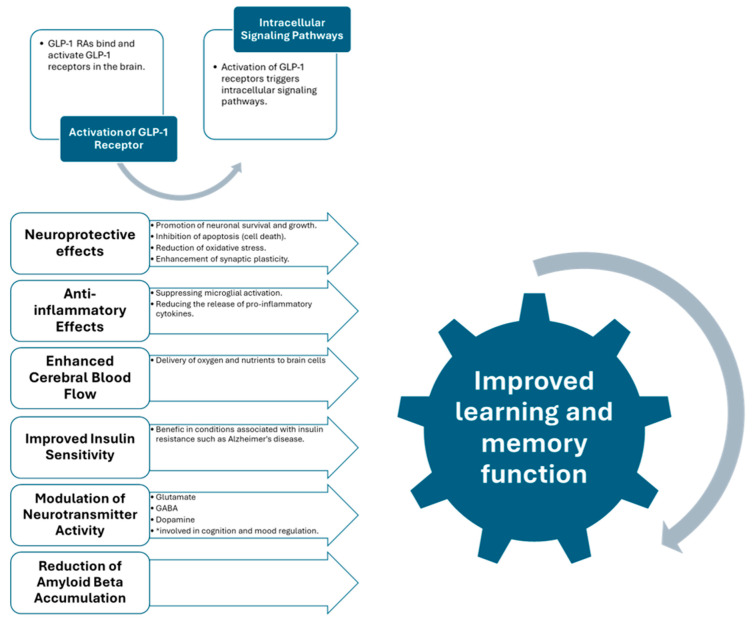
GLP-1 RAs and their protective role in cognition [1,2,3,4,5].

Various structures in the body, including the gastrointestinal tract, heart, lung, kidney, and brain, express glucagon-like peptide-1 receptors (GLP-1Rs) [1]. These findings suggest that GLP-1Rs could successfully treat diseases other than type 2 diabetes mellitus (T2DM) [2]. The widespread distribution of GLP-1 receptors in the brain specifies that GLP-1 signaling plays a wide range of roles in regulating more than a few physiological functions, including appetite control, glucose metabolism, cardiovascular function, mood regulation, and cognitive function [3]. Evidence suggests that GLP-1R agonists positively affect neurons, promote neurogenesis, reduce apoptosis and oxidative stress, and decrease neuroinflammation in many neurological disorders [4]. A schematic explanation of GLP-1 agonists’ effect on learning and memory improvement is provided in Figure 1.

Emerging research suggests GLP-1 RAs could help prevent neurological complications in individuals with diabetes, including stroke, cognitive impairment, and peripheral neuropathy [5]. A comprehensive analysis of 19 clinical trials proved outstanding cognitive improvements in patients with type 2 diabetes who were administered exenatide, dulaglutide, or liraglutide [6]. This emphasizes the potential therapeutic effect of GLP-1 RAs in improving overall cognitive function among people facing the challenges of diabetes [7]. Moreover, a separate study revealed that the treatment with GLP-1 RAs improved general cognition and positively impacted olfactory function in individuals with type 2 diabetes who were also challenging obesity [8]. These improvements in both cognitive and olfactory functions highlight the complex benefits GLP-1 RAs could bring to various neurological diseases [9]. These findings highlight their potential as an intriguing therapeutic strategy, offering a targeted approach to addressing neurological complications and a broader positive influence on cognitive and sensory functions [10]. Additionally, the benefits observed in numerous neurological illnesses suggest a promising trajectory for GLP-1 RAs in contributing to comprehensive care for individuals passing through the complex intersection of two significant diagnoses—diabetes and neurological challenges [11,12].

GLP-1 and its more stable, longer lasting analogs have been shown to have neuroprotective and anti-apoptotic effects. They can reduce beta-amyloid (Aβ) plaque accumulation and promote the distinction of neuronal precursor cells [12,13]. Available GLP-1 treatments also have a promising metabolic profile, including weight loss and reduced risk for hypoglycemia. Systematic evaluation of GLP-1 therapy’s effects in psychiatric populations who experience cognitive deficits represents a promising treatment path [14,15].

Insulin plays a central role in energy metabolism, gene expression, cell growth and repair, synaptic activity, apoptosis inhibition, and other essential processes that keep neurons healthy and efficient. The risk of developing neurodegenerative disorders increases if insulin signaling is frequently impaired in the brain [16]. Progressive neurodegenerative diseases and stroke bring, as a response, chronic inflammation in the brain, which leads to additional costs by triggering immune cells like microglia. Also, studies have shown that GLP-1 mimetics have anti-inflammatory properties [17,18]. Several studies proved that activated microglia and astrocytes, which participate in the immune/inflammation response, induced GLP-1 receptor expression. Also, GLP-1 treatment prevented an endotoxin-induced release of Interleukine-1β by these cells [19,20].

The US Food and Drug Administration (FDA) has certified semaglutide for treating type 2 diabetes mellitus (T2DM) through weekly subcutaneous injections at 0.25, 0.5, and 1 mg and daily oral doses of 3, 7, and 14 mg. Additionally, it is FDA-approved for extended weight management with weekly subcutaneous doses of 1.7 and 2.4 mg. Its mechanism involves increasing insulin release and decreasing glucagon secretion to regulate blood sugar levels [17]. There is also a particular focus on the effects of semaglutide, mainly on obesity. Beyond its established role in glycemic control, there is an increased interest in semaglutide’s potential influence on cognitive functions. Several studies have explored its impact on cognitive functions, with design, methodology, and result variations [18,19]. Antidiabetic medications are being considered for managing Alzheimer’s and Parkinson’s diseases due to the possible connection with diabetes mellitus. Evidence suggests that GLP-1 receptor agonists, like semaglutide, might offer neuroprotective qualities, potentially enhancing cognitive functions [11,12]. However, the findings are not entirely uniform.

Semaglutide’s effects on neuroinflammation, neurodegeneration, and oxidative stress, as quantified by biomarkers like C-reactive protein levels, provide the scientific rationale for selecting it as the focal point of our investigation. Nevertheless, no human studies were found that precisely evaluate its impact on cognitive function. Given the current research interest in potential effects on attention, memory, improvement of poststroke symptoms, or central nervous system infections, this systematic analysis aims to review existing studies that assess the impact of semaglutide, a newer GLP-1 agonist, on cognitive function.

## 2. Materials and Methods

### 2.1. Search Strategy

Our literature search was conducted on two online databases, MEDLINE (via PubMed) and Web of Science Core Collection, as well as various websites to retrieve all relevant articles published up to February 2024. The following keywords were used for this search: “semaglutide AND (cognition OR cognitive OR memory OR neurological OR brain function OR neuroprotection OR neurodegenerative)”.

### 2.2. Study Selection and Data Extraction

**Figure 2:**
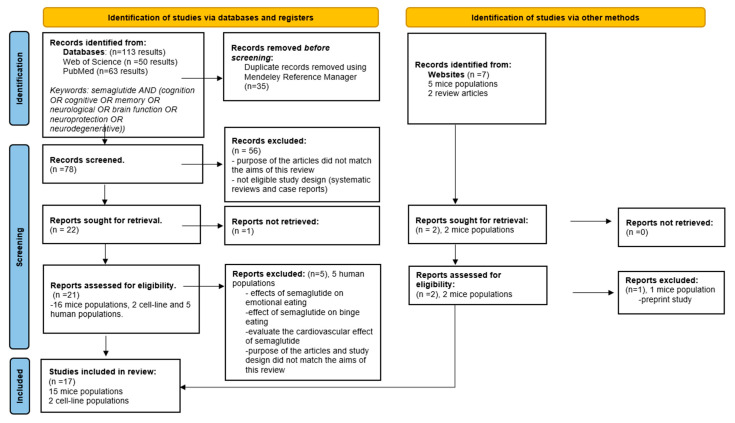
PRISMA flow diagram for search outcomes and screening process [21].

Titles, abstracts, and full-text articles were studied during the manual selection process to determine their relevance to the subject. Two authors independently divided the saved articles based on predefined inclusion/exclusion criteria. Any inconsistencies in the assessment were fixed through discussion with a third author. The entire selection procedure followed the guidelines outlined by PRISMA, as illustrated in the comprehensive flow diagram in Figure 2. Duplicates were removed automatically by the Mendeley Reference Manager [21]. Supplementary Files S2 and S3 on the PRISMA 2020 checklist can be consulted. 

#### 2.2.1. Inclusion Criteria

Studies were considered to be suitable for inclusion in this review if they met all of the following criteria: (1) The title included the keywords searched; (2) Titles were at least related to the keywords; (3) Articles written in English or articles written in other languages with abstracts or full text translated into English; (4) Abstracts available; (5) Full text available; (6) Minimum outcomes and statistical analysis; and (7) Articles of original research on semaglutide’s cognitive effects.

#### 2.2.2. Exclusion Criteria

Articles were excluded from the present study if they met any of the following criteria: (1) Inappropriate study design/article format; (2) Study not related to the cognitive effects of semaglutide; (3) No abstract available; (4) No full text available in English; and (5) Preprint study.

After meeting the eligibility criteria, the articles were referred to a detailed full-text analysis. This comprehensive examination included various aspects such as the objective of each study, the number of animal models used, the interventions applied, and the main results. 

### 2.3. Quality Assessment and Risk of Bias

**Figure 3:**
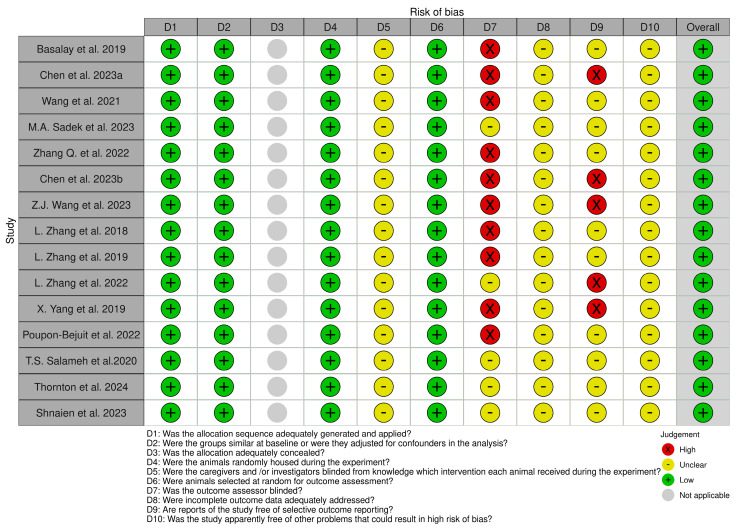
SYRCLE’s risk of bias summary [22,23,24,25,26,27,28,29,30,31,32,33,34,35,36,37,38,39].

**Figure 4:**
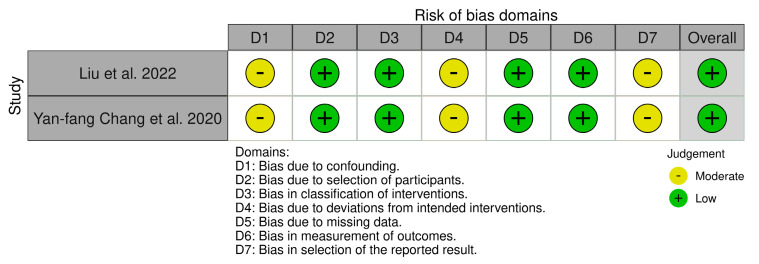
ROBINS-I Summary (risk of bias in nonrandomized studies of interventions) [22,23,24,40,41].

We used the Systematic Review Centre for Laboratory Animal Experimentation (SYR-CLE’s) RoB online tool to assess potential bias for animal models in 15 studies. The resulting visualizations, representing the risk of bias, are represented graphically in Figure 3 for greater clarity and accessibility. Also, the risk of bias for cell-line studies was assessed using the ROBINS-I online tool (risk of bias in nonrandomized studies of interventions), as represented in Figure 4 [22,23,24]. Also, Supplementary File S1 (Tables S1 and S2) on quality assessment and risk of bias can be consulted. 

## 3. Main Results

Our initial search yielded 113 potentially relevant articles from PubMed and Web of Science and seven studies from websites. The Mendeley reference manager automatically removed 35 duplicates.

In the screening phase, 78 articles were analyzed by title and abstract, and 56 were excluded due to missing abstracts, no eligible study design, or because the articles’ overall aim did not match the purpose of this review. The full text was reviewed for a final decision if the abstract did not provide enough information to clarify its relevance.

As a result of these steps, 21 articles from databases and four from websites underwent eligibility assessments. Five clinical studies were excluded from this review as they did not specifically investigate the effects of semaglutide on cognition, and one study was excluded because of preprint status [42,43,44,45,46]. Finally, 17 articles were eligible, including 15 mice populations and two cell lines, followed by a thorough analysis of their full texts. Table 1 contains a synthesis of the included studies, which offers an easy-to-read reference for readers to interpret the findings conveniently. We have registered our systematic review protocol in OSF Registries and the published version is available on https://osf.io/n3hxf (accessed on 23 April 2024). 

## 4. Discussion

### 4.1. Semaglutide, a State-of-the-Art Synthetic GLP-1 Analog

Semaglutide is a multifaceted agent that has shown efficacy in reducing apoptosis, enhancing neuroprotection, managing metabolic processes, and improving cognitive function [29]. New aspects of this synthetic GLP-1 agonist impact have been highlighted, showing that it can potentially facilitate cognitive dysfunction associated with cerebrovascular diseases. These noteworthy findings identify semaglutide as a versatile therapeutic agent with promising neuroprotective implications in the broader context of cerebrovascular disease management [30].

The neurocognitive impact of T2DM suggests a marked acceleration of normal brain aging, and T2DM increases the risk for AD by approximately twofold [27]. According to a study by Shanxi Medical University in China, semaglutide may have neuroprotective effects against β-amyloid toxicity and prompts further investigations into the complex molecular pathways involved in AD models [31]. The positive effect of semaglutide is suitable for its ability to improve autophagy and inhibit apoptosis. However, it is essential to note that this study was conducted in vitro, warranting further exploration to determine its efficacy in vivo [40]. Considering the deficit of other systematic reviews on this specific subject, the study conducted by Shanxi Medical University stands as a unique assessment exploring literature on semaglutide’s cognitive effects [31,41]. A notable aspect of this analysis is its exclusive focus on rodent studies that met specific eligibility criteria. Evolution to clinical studies is essential for semaglutide’s effectiveness and safety as a cognitive supplement [47,48]. While the protective effects against β-amyloid toxicity observed in controlled laboratory settings are promising, the real-world efficacy and potential side effects must be rigorously examined [48,49]. The ability of semaglutide to cross the blood-brain barrier {BBB} and modulate brain-derived neurotrophic factor (BDNF) signaling may contribute to its neuroprotective effects [50,51,52].

Even if some articles’ overall aim did not match the purpose of this review and were excluded, there is essential information about the implications of synthetic GLP-1 agonists. In 2024, a study was conducted to examine the impact of semaglutide on obese patients associated with heart failure and revealed its positive effect on functionality and quality of life [42]. Also, the results of the retrospective cohort study led by J. Richards et al. provide compelling evidence supporting the efficacy of semaglutide in the treatment of binge eating disorder [43]. J. Nicolau et al. also provided valuable insights into its short-term effects on abnormal eating patterns, including emotional eating, among individuals with obesity [44]. Led by LM Pérez-Belmonte et al., the real-world study investigated the efficacy and safety of semaglutide in managing obese patients with type 2 diabetes (T2D) and chronic heart failure (CHF). Participants receiving this treatment experienced improvements in glycemic control, indicating its effectiveness as an adjunctive therapy for T2D management in obese individuals with CHF [45]. It is noted that the feasibility study conducted by T. Davidy et al. aimed to provide the basis for a multicenter, large-scale, randomized clinical trial of combining intranasal insulin with dulaglutide for improving cognition in older adults with metabolic syndrome who are at high risk of developing dementia [46].

Rodent model studies have consistently demonstrated that semaglutide benefits cognitive function, manifested by improved memory, learning, and overall mental performance. Also, the Phase 3 clinical trial EVOKE aims to assess the safety and efficacy of orally administered semaglutide in the early stages of Alzheimer’s Disease [53,54,55].

### 4.2. Cell Line Studies

Derived from human neuroblastoma, the SH-SY5Y cell line can differentiate into cells similar to neurons and represents a fundamental tool for exploring pharmacological compounds’ neuroprotective properties. Our systematic review included two studies on cell lines. In 2020, Chang et al. investigated how semaglutide treatment affected amyloid-beta (Aβ) pathology and explored the underlying cellular mechanisms involving autophagy and apoptosis. By utilizing SH-SY5Y cells impaired by Ab_25–35_, a specific sequence of amino acids derived from beta-amyloid peptides, semaglutide treatment enhanced autophagy. This cellular process that clears misfolded proteins, including Aβ, could facilitate the removal of toxic protein aggregates and mitigate neurodegeneration in AD [41].

Two years later, Liu et al.’s study focused on the protective effects of semaglutide against 6-hydroxydopamine (6-OHDA) toxicity in a model of Parkinson’s disease (PD). Additionally, the impact of semaglutide and liraglutide at the same dose was compared. Both compounds were discovered to protect against 6-OHDA cytotoxicity in SH-SY5Y cells by improving autophagy flux, reducing oxidative stress, and mitigating mitochondrial dysfunction [40].

These two cell-line studies propose that semaglutide could act as a disease-modifying therapy, potentially delaying disease progression and preserving motor function in Parkinson’s disease (PD) patients. Also, it may inhibit apoptosis in neuronal cells affected by Aβ pathology, thereby potentially preserving neuronal function and cognitive abilities in AD patients. Additionally, its therapeutic potential in Parkinson’s disease lies in its ability to protect dopaminergic neurons from damage [40,41].

### 4.3. Rodent Studies

The following 15 studies were conducted on rodents and have been classified as follows: [25,26,27,28,29,30,31,32,33,34,35,36,37,38,39]

- Three studies analyzed the effects of semaglutide on ischemic stroke [25,29,35].
- Five studies analyzed neurodegenerative diseases (one on Alzheimer’s disease, three on Parkinson’s disease, and one that analyzed both Parkinson’s and Alzheimer’s diseases together) [31,32,33,34,37].
- Three studies analyzed the effect of semaglutide on high-fat-induced mice [26,30,38].
- Four studies analyzed separately various inflammatory models, including inflammation, epilepsy, multiple sclerosis, and infantile neurometabolic disease [27,28,36,39].

#### 4.3.1. Semaglutide and Ischemic Stroke

Managing cognitive symptoms among stroke survivors is essential to optimize their functional rehabilitation and improve their overall quality of life [56]. Basalay et al. evaluated the neuroprotective effects of liraglutide and semaglutide using a nondiabetic rat model of acute ischemic stroke. Yang et al.’s study focused on assessing the impact of semaglutide alone on infarct size, inflammation, apoptosis, and neurogenesis in mice following stroke. Both studies involved middle cerebral artery occlusion (MCAO) [25,35].

The neuroprotective effects of liraglutide and semaglutide involve potential applications in neurological conditions characterized by ischemia−reperfusion injury and administered subcutaneously, reduced infarct size by 63% and 48%, respectively. Also, in the semaglutide-treated group, all rodents survived for 72 h and no significant intracerebral hemorrhages were detected. Liraglutide’s infarct-limiting and functional neuroprotective effects are dose-dependent, and the principal clinic-related difference between semaglutide and liraglutide is the substantially longer half-life of the first, according to Basalay et al. [25,56].

In the same year, Yang et al.’s study observed the brain’s neurogenesis and inhibited apoptosis following semaglutide treatment. Decreasing infarct size in rats following a stroke suggests that semaglutide may have a protective effect on brain tissue, potentially limiting the extent of damage caused by ischemia−reperfusion injury. The GLP-1 treatment improved motor control and muscle strength without affecting blood glucose levels. Semaglutide’s multifaceted effects on reducing infarct size, inflammation, apoptosis, and promoting neurogenesis highlight its potential as a neuroprotective agent in stroke [35].

Three years later, Zhang and colleagues assessed the impact of A1 astrocytes on blood-brain barrier (BBB) dysfunction following stroke, using mice subjected to transient middle cerebral artery occlusion (TMCAO). The study focused on blocking the conversion of C3d+/GFAP+ A1 astrocytes using semaglutide. A1 astrocytes contribute to neuroinflammation and neuronal damage, and blocking their conversion may mitigate the inflammatory response and promote neuroprotection. According to Zhang et al., semaglutide treatment reduced BBB disruption in mice following ischemic stroke, suggesting that inhibiting astrocyte conversion offers a new perspective in ischemic stroke therapy [29,56]. There is significant evidence to support the use of semaglutide in managing post-stroke symptoms, but more human studies are needed in this area of research.

#### 4.3.2. Semaglutide and Neurodegenerative Diseases

Due to a shared pathogenesis involving insulin resistance, type 2 diabetes mellitus (T2DM) represents a significant risk factor for Alzheimer’s disease (AD). As a result, antidiabetics have shown the potential to slow down AD progression. Glucagon-like peptide one receptor agonists (GLP-1RA) can diminish neuroinflammation and oxidative stress and reduce AD models’ amyloid-beta deposition or tau hyperphosphorylation [57,58].

Amyloid plaques, neurofibrillary tangles, neuroinflammation, and oxidative stress are the key features that characterize Alzheimer’s disease (AD) at the cerebral level. Amyloid-beta (Aβ) plaques are considered foreign material in the brain, which triggers an inflammatory and immune response through microglia activation. This chain of events ultimately results in neuronal degeneration and synaptic damage [58,59].

In normal circumstances, acute inflammation is naturally resolved through the anti-inflammatory effects of microglia. In AD, amyloid deposits persist, leading to chronic neuroinflammation, exacerbating neurodegeneration, and triggering immune cell infiltration through the blood-brain barrier [59].

Insulin injection into the hippocampus and intranasal administration of insulin improves spatial memory. Abnormal lipid metabolism increases the risk of amyloid pathology development in AD brains. GLP-1 and its mimetics can cross the blood-brain barrier and affect cognition and neuroprotection. Researchers in endocrinology, neurology, and psychiatry should collaborate to explore the therapeutic potential of semaglutide in preserving cognitive function and mitigating neurodegenerative diseases [52,60].

Previously, studies have demonstrated that semaglutide shows neuroprotective characteristics in animal models of both Parkinson’s disease and stroke. In 2023, Wang et al. investigated the effect of semaglutide on cognitive function and glucose metabolism in a model of Alzheimer’s disease in the 3xTg mouse. They studied the role of the GLP-1R/SIRT1/GLUT4 pathway [31].

To understand the study’s protocol, we mention that Sirtuin 1 (SIRT1) is a protein that regulates cellular processes, including metabolism, immune response, and aging. Research indicates that the SIRT1 family improves neurodegenerative diseases and represents a promising target for therapeutic interventions in managing type 2 diabetes. GLUT4, a variant of the GLUT protein family, is a glucose transporter protein primarily found in adipose tissue and skeletal muscle cells, which adjusts glucose metabolism in association with insulin [31]. Studies indicate that GLP-1 alleviates palmitic acid-induced insulin resistance in human skeletal muscle by modulating SIRT1 and GLUT4 function through diverse molecular mechanisms. Wang et al. identified significant overexpression of SIRT1 and GLUT4 in the CA3 region, recognized as one of the hippocampus’s most functionally significant areas. Semaglutide improves memory in AD rodent models by activating the GLP-1/SIRT1/GLUT4 pathway and facilitating glycolysis [40].

Parkinson’s disease is one of the neurodegenerative conditions extensively investigated regarding its pathogenic mechanisms. While both studies by Zhang et al. focus on the neuroprotective properties of semaglutide in Parkinson’s disease (PD) mouse models induced by 1-methyl-4-phenyl-1,2,3,6-tetrahydropyridine (MPTP), they differ in their specific objectives and findings [32,33].

In 2018, Zhang and colleagues evaluated semaglutide’s neuroprotective effect compared with liraglutide, which was given at the same dose. A year later, they examined the impact of semaglutide on α-synuclein levels, an essential protein associated with PD pathology, making the same comparison between the two GLP-1 agonists. The study found that semaglutide wielded neuroprotective effects and reduced α-synuclein levels, suggesting a potential mechanism for its therapeutic action in PD [33,40].

It is believed that the accumulation and aggregation of α-synuclein is caused by its overexpression and the failure to clear the protein through proteolysis and autophagy mechanisms efficiently. Semaglutide treatment decreased α-synuclein levels in the mice brains and maintained a balance of dopaminergic neurons in the substantia nigra pars compacta. The progressive loss of dopaminergic neurons reduces TH levels and subsequently diminishes dopamine production. According to Zhang et al.’s results, both drugs’ ability to release TH levels suggests their potential as therapeutic interventions for Parkinson’s disease. Restoring TH levels may help to maintain or even improve dopamine production, which could improve symptoms associated with dopamine deficiency, such as movement difficulties and motor fluctuations [26,32,33].

Another study, published in 2022, analyzed the neuroprotective effects of two compounds, DA5-CH and semaglutide, in a 6-hydroxydopamine (6-OHDA) rodent model of Parkinson’s disease. In contrast to the MPTP model, 6-OHDA shows a reduced number of neurons in the substantia nigra, suggestive of neurodegeneration. Also, DA5-CH is a dual GLP-1/GIP receptor agonist that can cross the blood-brain barrier faster than semaglutide. Specifically, the researchers investigated whether these compounds could protect against neurodegeneration and reduce the levels of α-synuclein. After drug treatment, a decrease in α-synuclein levels was noted, with DA5-CH exhibiting greater efficacy in reducing these levels compared to semaglutide [34].

Research has shown that certain incretins, such as glucagon-like peptide-1 and glucose-dependent insulinotropic polypeptide, interfere with brain regions implicated in Alzheimer’s disease (AD) and Parkinson’s disease (PD). There are two types of incretin receptor agonists (IRAs): single IRAs, which target receptors for a single kind of incretin, and dual IRAs, which target receptors for both types. The GLP-1 receptor agonists (GLP-1RAs) are single IRAs that have shown promise as potential therapeutics for AD and PD. These include exendin-4, liraglutide, lixisenatide, and semaglutide, which are FDA-approved. Dual IRAs also have potential as therapeutics for these disorders, but they still need to be assigned generic names [34,37]. According to Salameh et al.’s study from 2020, regardless of the positive effects of semaglutide at the cerebral level, it fails to penetrate the blood-brain barrier when evaluated for the entire brain. Within 10 min of intravenous administration, exendin-4 and lixisenatide present significant rates of blood-to-brain influx, while liraglutide and semaglutide do not. These findings suggest that acylated IRAs, liraglutide and semaglutide, cannot cross the blood-brain barrier [37].

It is also important to mention that some of the most recent studies, published at the beginning of 2024, analyze innovative mechanisms in the context of neurodegenerative diseases. Researchers explored how mutated E3 ligase affects cellular stress responses and contributes to neurodegeneration, providing novel insights into the pathophysiology of neurodegeneration and stress response regulation at the cellular level. E3 ligases are enzymes that play a crucial role in the ubiquitin−proteasome system, which targets proteins for degradation [61]. In January 2024, Haakonsen et al. investigated how dysfunctional E3 ligases may transform cells into more susceptible ones to stress-induced damage. Also, in March 2024, Haney and colleagues explored the association between the APOE4/4 genotype and lipid droplets in microglia cells in Alzheimer’s disease (AD). It was suggested that individuals with the APOE4/4 genotype present an increased accumulation of damaging lipid droplets within microglia cells [62].

#### 4.3.3. Cognitive Impairment and Obesity

Recent preclinical and clinical research findings have revealed lower cognitive performance in executive function, memory, inhibition, and language domains associated with obesity. Furthermore, increased blood-brain barrier permeability permits peripheral pro-inflammatory markers to diffuse in the brain, inducing neuroinflammation. The following three studies examine the effects of semaglutide on high-fat-induced mice: two studies from 2023 and one from 2024 [26,30,38].

Chen et al. studied the effect of semaglutide and empagliflozin treatment on phosphorylated protein expression in the hippocampus of obese mice using the phosphorylated 4D-LFQ technique. Protein phosphorylation is vital for eukaryotic signaling, regulating processes like cell cycle, development, and metabolism. 4D-LFQ proteomics technology isolates proteins and uses mass spectrometry and bioinformatics for precise results. Semaglutide and empagliflozin improved cognitive function compared to untreated obese mice, resulting in decreased escape latency, an increased percentage of swim time in the target quadrant, and an enhanced frequency of passing through the platform area, with minimal differences between the effects of the two drugs [26].

The phosphorylation patterns of specific proteins in the hippocampus were analyzed to investigate the cognitive effects of semaglutide and empagliflozin. In mice, a diet high in fat can have a negative impact on cognitive function by decreasing the phosphorylation levels of proteins involved in the dopaminergic synapse pathway. Reduced phosphorylation can interrupt signaling pathways for cognitive function, impairing learning and memory processes. It is believed that semaglutide and empagliflozin can improve cognitive function by activating the dopaminergic synapse pathway. This activation may promote the phosphorylation of CACNA1D, CACNA1A, and CACNA1B, genes implicated in the normal functioning of the nervous system. As a result, cognitive deficits caused by a high-fat diet-induced impairment can be prevented or improved [30].

Semaglutide can help maintain a standard neurocytoskeletal structure, improve axon-al growth, and stimulate neurogenesis by reducing the phosphorylation of HTT (Huntingtin) and NEFH (neurofilament heavy polypeptide) and increasing the phosphorylation of NEFL (neurofilament light polypeptide). This mechanism can reduce the risk of cognitive impairment caused by obesity [38].

Thornton and colleagues examined the effects of two NLRP3 inflammasome inhibitors (NT-0249 and NT-0796) on obesity-induced complications such as systemic inflammation and astrogliosis. Chronic inflammation is a common characteristic of obesity, and inhibiting the NLRP3 inflammasome may help reduce this inflammatory response. The results showed a decrease in astrogliosis, which suggests that NLRP3 inflammasome inhibition could have neuroprotective effects. This implies that NLRP3 inflammasome inhibitors may have a broader therapeutic potential beyond metabolic disorders, extending to neurological conditions associated with neuroinflammation [26,30,38].

#### 4.3.4. Semaglutide and Inflammatory Diseases

Inflammation is a significant factor in numerous neurological disorders. For example, multiple sclerosis (MS) is characterized by inflammatory demyelinated lesions that affect white and gray matter. In addition, several neurological conditions, such as Huntington’s or Alzheimer’s disease, vascular dementia, brain tumors, and autism, are frequently associated with abnormal synchronized neuronal activity [63].

Frequent seizures, particularly status epilepticus, induce oxidative stress, alterations in growth factors like BDNF, and inflammation within the brain. It has been shown that untreated seizures may cause long-term cognitive deficits, and around 60–70% of individuals with chronic epilepsy experience cognitive dysfunction [64].

As a pathophysiological explanation for the relationship between epilepsy and cognition, seizures and epileptiform discharges observed in EEG are proposed to directly damage neural networks essential for cognitive function. Also, evidence indicates that the hippocampal areas CA1 and CA3 are especially susceptible to neuronal damage in individuals with epilepsy [65].

In addition to seizures, epilepsy patients may experience cognitive dysfunction, including memory, attention, and information-processing deficiencies [66]. Evidence has shown that neuroinflammation plays a central role in the pathological mechanism of epilepsy. The NLRP3 inflammasome is a cluster of proteins that triggers an inflammatory response and promotes the release of pro-inflammatory cytokines [27]. In 2021, Wang et al. investigated the effects of semaglutide on seizure severity and cognitive dysfunction in a rodent model of epilepsy induced by pentylenetetrazole (PTZ) kindling. The study established an in vitro inflammatory model using lipopolysaccharide and nigericin stimulation in BV2 cells. Also, chronic epilepsy model mice were generated using the PTZ kindling method. Semaglutide decreased NLRP3 inflammasome activation and the secretion of inflammatory cytokines. Its inhibitory effect in both vivo and in vitro models mitigated neuronal apoptosis, improved cognitive function, and reduced seizure severity [26,27].

Emerging research has revealed that the development of multiple sclerosis may be significantly linked to inflammation in the nervous system and oxidative stress. Glycogen synthase kinase-3 beta (GSK-3β) is a vital protein kinase known to be upregulated in various neurological disorders, including chronic progressive MS. Therefore, targeting GSK-3β may be a promising strategy for developing new therapies for MS [28].

Sadek and colleagues recently investigated the potential therapeutic effects of semaglutide on experimental autoimmune encephalomyelitis (EAE)-induced multiple sclerosis (MS) in mice. The EAE animal model often replicates significant aspects of MS. It has been found that semaglutide has a stimulating effect on the phosphoinositide 3-kinase/protein kinase B/glycogen synthase kinase-3 beta pathway in the central nervous system (CNS) in mice. Activation of this pathway is associated with anti-inflammatory and neuroprotective effects, which may contribute to the therapeutic benefits of semaglutide in MS [27,28].

Semaglutide treatment reduced CNS inflammation, as evidenced by decreased immune cell infiltration and reduced levels of pro-inflammatory cytokines in the spinal cords of EAE-induced MS mice. In addition to its anti-inflammatory properties, this new GLP-1 agonist may prevent MS-related demyelination and neurodegeneration, according to recent studies [28].

Infantile neuroaxonal dystrophy (INAD) is a rare and progressive condition found in children stemming from mutations in the PLA2G6 gene. This gene mutation is also responsible for PARK14-linked dystonia-parkinsonism, which manifests in young adults. While the main features of INAD include developmental and motor delays, cognitive impairment is also commonly observed in affected individuals [67].

In 2022, Poupon-Bejuit et al. investigated for the first time the therapeutic effects of semaglutide on neurodegeneration in a mouse model of infantile neurometabolic disease. Administering semaglutide at a high weekly dose to young INAD mice improved locomotor function and increased lifespan [10,11]. Additionally, there was a significant decrease in the expression of mediators involved in apoptotic pathways in mice treated with semaglutide. Furthermore, a reduction in neuronal loss and neuroinflammation was observed. Semaglutide slows neurodegeneration caused by infantile neurometabolic disease by promoting neuronal survival in the brain and reducing inflammation and oxidative stress [36].

Various mechanisms have been suggested to contribute to the long-term cognitive decline following sepsis, including disruption of the blood-brain barrier, neuroinflammation, dysfunction of neurotransmitters, and neuronal loss [68]. Targeting these essential processes could effectively prevent and treat cognitive impairment associated with sepsis or endotoxemia, even if the pathophysiological mechanism of this association remains unclear mainly [69].

Regarding bacterial toxins in the bloodstream, endotoxemia leads to systemic inflammation and neuroinflammation, ultimately resulting in neurological damage. According to Shnaien et al. [39], semaglutide treatment reduced cellular injury in endotoxemia mouse brains, suggesting possible mitigation of neurologic damage caused by systemic inflammation.

While the preclinical findings and cell line studies are promising, further research, including clinical trials, is needed to validate the efficacy and safety of semaglutide in mitigating neurologic damage. Research has confirmed that semaglutide can be a versatile therapeutic agent for various neurological conditions. It includes epilepsy and cognitive dysfunction that are associated with obesity, stroke, and neurodegenerative diseases. This conclusion is based on evidence gathered from animal models and cell line studies. Before conducting human trials, understanding how semaglutide affects animals’ neural pathways and cognitive function is essential. Cell line and animal studies inform semaglutide’s potential as a therapeutic agent for mental disorders in humans, contributing to the translational process from preclinical research to clinical applications.

### 4.4. New Insights—Gut-Brain Axis Impact on Cognition

This intricate gut−brain axis relationship is mediated through the autonomic nervous system and the hypothalamic−pituitary−adrenal axis. Perturbations in the gut microbiota have been implicated in anxiety and depression. Stress, pervasive in contemporary society, has been shown to induce alterations in the intestinal microbiome, leading to intestinal dysbiosis [69,70]. Intestinal dysbiosis is associated with local and systemic inflammation, contributing to the pathogenesis of various diseases, including obesity, diabetes, and neurodegenerative disorders. Disruptions in intestinal integrity can cause bacteria and their by-products to enter the systemic circulation, leading to inflammation in the body and brain [71].

In Parkinson’s disease (PD), intracellular protein deposits known as Lewy bodies have been found in the enteric nervous system, implicating intestinal dysbiosis in the genesis of PD symptoms. The gut−brain axis is also involved in autism spectrum disorder (ASD), with many patients exhibiting gastrointestinal symptoms, possibly stemming from early alterations in gut colonization and antibiotic exposure [72].

Highlighting the significance of maintaining a healthy microbiota, dietary interventions aimed at reducing fat and sugar intake, increasing fiber consumption, and avoiding alcohol and smoking are advocated. Additionally, promoting physical activity and stress reduction is essential [72,73].

## 5. Conclusions

This systematic review synthesizes data from various studies to provide a comprehensive overview of semaglutide’s neuroprotective potential. The complex interaction between its effects on seizures, neuronal damage, cognitive function, and other neurobiological parameters paints a nuanced picture of its therapeutic promise. However, it is imperative to approach these findings with cautious optimism, recognizing the complexities of translating preclinical success into clinical efficacy.

Semaglutide emerges as a multifaceted compound with promising neuroprotective properties in rodent models. Its impact on seizures, neuronal damage, cognitive function, and other neurobiological aspects positions it as a candidate for further exploration in clinical settings. The potential role in stroke management, prevention of obesity-induced cognitive impairment, and protection against sepsis-induced brain dysfunction add layers to its therapeutic potential. While the current evidence is compelling, rigorous clinical research is warranted to validate these preclinical and in vitro findings and discover semaglutide’s safety and efficacy in humans.

The journey from preclinical promise to clinical application requires careful consideration. Still, the cumulative evidence suggests semaglutide may hold critical answers to address neurological conditions and improve cognitive status. Semaglutide has notably reduced the severity of seizures in rodent models. In addition to its impact on seizures and neuronal damage, semaglutide has demonstrated an ability to enhance cognitive function in animal models. This promising effect indicates its potential utility in addressing cognitive deficits associated with various neurological conditions.

Beyond its effects on seizures, neuronal damage, and cognitive function, semaglutide has shown promise in addressing spatial learning and memory dysfunction in obese mice. Also, identifying specific phosphorylated proteins affected by semaglutide could elucidate novel molecular targets for therapeutic intervention in obesity-related cognitive dysfunction. Semaglutide and empagliflozin may offer additional benefits beyond weight loss by positively impacting cognitive function, which is often impaired in obese individuals.

One notable benefit of semaglutide in animal models is its potential as a treatment for stroke. By decreasing the risk of cognitive impairment caused by obesity, semaglutide emerges as a promising option for repurposing in stroke management. The neuroprotective effects observed in rodent models suggest its ability to reduce the consequences of cerebrovascular events, making it an exciting avenue for further exploration in clinical settings. Clinical trials that evaluate these medications in patients with acute cerebrovascular events could provide valuable insights into their effectiveness and safety profiles in real-world scenarios.

Semaglutide also exhibits protective effects against sepsis, a systemic inflammatory response that can lead to severe complications, including brain dysfunction. Its role as a preventive measure for brain dysfunction in sepsis underscores its broad-ranging neuroprotective properties. The current understanding, primarily derived from preclinical studies, lays the groundwork for future investigations into the clinical efficacy and safety of semaglutide in the context of neurological disorders. Cell-line and animal studies have demonstrated its neuroprotective effects, but further research is needed to confirm these findings and assess the impact on human patients. Future investigations should focus on identifying the molecular mechanisms involved in semaglutide’s action on the brain and evaluating its effectiveness and safety in clinical trials. These efforts could contribute to developing innovative therapeutic strategies for preserving cognitive function and preventing neurological disorders, offering hope for improving patients’ quality of life.

This study has limitations: Only 17 articles were included, and research was conducted on only two online databases. No published results were found on semaglutide’s effects on individuals with cognitive deficits. Even if more research on this topic is needed, this systematic review presents an overview of semaglutide’s potential effects on animal models and cell lines in various pathologies that cause cognitive impairment.

## Figures and Tables

**Table 1:** Synthesis of included studies [25,26,27,28,29,30,31,32,33,34,35,36,37,38,39,40,41].

Scheme 1.	Aim	Animal Model/Cell Line	Methods	Results
1. Basalay et al. 2019 [25]	-Examine the Neuroprotective Effects of liraglutide And semaglutide	Male nondiabetic Sprague-Dawley rats	-A model of acute ischemic stroke (middle cerebral artery occlusion) was used to assess the effects of liraglutide or semaglutide administered i.v. at onset or s.c. before it. -Infarct size and functional status were evaluated after 24 or 72 h of reperfusion.	-Liraglutide reduced brain damage dose-dependent. -Reperfusion delay is a limiting factor For neuroprotection by liraglutide. -Neuroprotection by semaglutide is at least as potent as by liraglutide. -The principal clinic-related difference between semaglutide and liraglutide is the substantially longer half-life.
2. Chen et al. 2023 [26]	-Investigate differences in cognitive function and hippocampal phosphorylated protein expression in high-fat diet-induced obese mice after treatment with semaglutide and empagliflozin.	C57BL/6JC male mice	-Control (C) and high-fat diet (H) -After 12 weeks, Group H Was divided into: -Group H -Group semaglutide: Intraperitoneal injections of 30 nmol/kg/d bodyweight of Semaglutide for 12 weeks -Group empagliflozin: gavage administration of 10 mg/kg/d bodyweight of empagliflozin for 12 weeks -Groups C and H received equal volumes of saline via the same routes.	-The use of semaglutide treatment decreased escape latency and Increased the total percentage of time spent swimming. -Semaglutide and empagliflozin increased the phosphorylation of CACNA1D, CACNA1A, and CACNA1B proteins.
3. Wang et al. 2021 [27]	-Investigate the functions of semaglutide in epilepsy and inflammation models	C57BL/6J mice	-In vitro: inflammation model Using lipopolysaccharide (LPS) and nigericin stimulation in BV2 cells. -In vivo: chronic epilepsy model using a pentylenetetrazole (PTZ) kindling method.	-Semaglutide decreased inflammation and reduced cell damage by blocking NLRP3 inflammasome activation in BV2 cells treated with LPS and nigericin. -Semaglutide reduced seizure severity, prevented hippocampal neuron death, improved cognition, blocked NLRP3 inflammasome activation, and lowered inflammatory cytokine levels.
4. M.A. Sadek et al. 2023 [28]	-Investigate the Influence of semaglutide on experimental autoimmune encephalomyelitis (EAE)-induced multiple sclerosis (MS) in mice.	Adult male Swiss albino mice	-The standard control group received saline (0.2 ml; intraperitoneal [i.p.]). -The semaglutide-control group received semaglutide (25 nmol/kg/day; i.p.) -The EAE group was subjected to EAE induction and treated with saline (0.2 ml; i.p.). -The semaglutide-treated group was subjected to EAE induction and treated with semaglutide (25 nmol/kg/day i.p.) Two hours after EAE induction. -All treatments were administered for two weeks starting from EAE induction.	-Semaglutide: reduced EAE-induced clinical signs -Mitigates EAE-induced weight changes in CNS organs -Attenuated EAE-induced motor impairment, cognitive dysfunction -Amended EAE-induced histological alterations in the corpus callosum and brain atrophy and demyelination -Lessened EAE-induced oxidative stress and neuroinflammation
5. Zhang Q. et al. 2022 [29]	-Explore the effect of A1 astrocytes on BBB integrity after ischemic stroke	Adult male ICR mice	-Transient middle cerebral artery occlusion (TMCAO). -Immunohistochemical staining of A1 (c3d) and A2 (S100A10) were performed to characterize astrocyte phenotypic changes after TMCAO. -Intraperitoneal injection with semaglutide to inhibit A1 astrocytes.	-The number of c3d+/GFAP+ A1 astrocytes increased within 14 days after S100A10+/GFAP+ TMCAO, while A2 astrocytes decreased. -Treatment with semaglutide decreased the count of microglia expressing CD16/32 and astrocytes expressing C3d and GFAP, which are characteristic of A1 astrocytes. -Semaglutide reduced brain infarct volume and neuroinflammation; improved neurobehavioral outcomes.
6. Chen et al. 2023 [30]	-Investigate the effects of semaglutide on phosphorylated protein expression and its neuroprotective mechanism in hippocampi of high-fat-diet- induced obese mice	C57BL/6J male normal mice	-Control group (c) regular diet -Model group (H) high-fat diet + saline -Semaglutide group (H+ semaglutide, group S) semaglutida 30 nmol/kg/d for 12 weeks -Morris water maze assay to detect cognitive function changes -Phosphorylated proteomic analysis to detect the hippocampal protein profile in mice	Semaglutide intervention on high-fat diet-induced obese mice: -reduced body weight -improved oxidative stress indexes -shortened the water maze platform latency -Semaglutide reduces phosphorylation and increases neuroprotection in obese mice.
7. Z.J. Wang et al. 2023 [31]	-Correlate the GLP-1R/SIRT1/GLUT4 pathway with glucose metabolism. -Investigate the mechanism of action For improving glucose metabolism in the 3xtg transgenic mouse model of Alzheimer’s disease and cultured neurons.	-male APP/PS1/Tau transgenic mice (3xtg) -C57B6/129 wild-type mice (WT)	-WT + saline group -3xtg + saline group -3xtg + semaglutida group -3xtg + semaglutide + EX527 (SIRT1 inhibitor) group -15 mice in each group were injected with semaglutide (0.1 mg/kg, i.p.) or saline (0.9%, i.p.) every other day for 30 days before the behavioral experiment.	Semaglutide increased the expression levels of SIRT1 and GLUT4 in the hippocampus of 3xtg mice, → improved learning and memory, and decreased Aβ plaques and neurofibrillary tangles. -Improved glucose metabolism, promoted glycolysis, and improved glycolytic disorders -Increased the membrane translocation of GLUT4 in cultured HT22 cells.
8. Liu et al. 2022 [40]	-Explore the neuroprotective effects of semaglutide in PD -Compared the effect of semaglutide with liraglutide at the same dose	-SH-SY5Y The cell line of human neuroblastoma	-Treated the human neuroblastoma SH-SY5Y the cell line with 6-hydroxydopamine (6-OHDA) as a PD in vitro model	-Semaglutide and liraglutide protect against 6-OHDA cytotoxicity by increasing autophagy flux and decreasing oxidative stress and mitochondrial dysfunction in SH-SY5Y cells. -Semaglutide was superior to liraglutide for most parameters measured
9. L. Zhang et al. 2018 [32]	-Report the protective effects of semaglutide (25 nmol/kg ip, once daily for seven days) in the MPTP mouse model of PD. -Compare the neuroprotective effects of semaglutide and liraglutide when given at the exact dosage.	-Male C57BL/6 Mice	-Control group treated with saline -Liraglutide group treated with saline and liraglutide -Semaglutide group treated with saline and semaglutide -MPTP group treated with MPTP -MPTP (once daily 20 mg/kg i.p. for seven days) followed immediately by liraglutide-treated group (25 nmol/kg i.p. once daily for seven days) -MPTP (20 mg/kg i.p. once daily for seven days) followed immediately by semaglutide-treated group (25 nmol/kg i.p. once daily for seven days).	-Semaglutide and liraglutide were found to have improved the motor impairments caused by 1-methyl-4-phenyl-1,2,3,6-tetrahydropyridine (MPTP). -Semaglutide was found to be better than liraglutide in most of the measurements taken.
10. L. Zhang et al. 2019 [33]	-Investigate the neuroprotective effects of semaglutide (25 nmol/kg i.p. once every two days for 30 days) and liraglutide (25 nmol/kg i.p. once daily for 30 days) in the chronic MPTP mouse model of PD.	-Male C57BL/6 Mice	-Control group treated with saline -Liraglutide group (25 nmol/kg i.p. once daily for 30 days) -Semaglutide group (25 nmol/kg i.p. once every two days for 30 days) -MPTP group treated with MPTP alone (once daily 20 mg/kg i.p. for 30 days) -MPTP (once daily 20 mg/kg i.p. for 30 days) + liraglutide-treated group (25 nmol/kg i.p. once daily for 30 days) -MPTP (20 mg/kg i.p. once daily for 30 days) + semaglutide-treated group (25 nmol/kg i.p. once every two days for 30 days).	MPTP-induced increase in alfa-syn expression in the brain is reduced by semaglutide and liraglutide. Semaglutide is more potent than once daily liraglutide in most parameters measured.
11. L. Zhang et al. 2022 [34]	-Developed a dual GLP-1/GIP receptor agonist (DA5-CH) that can cross the blood-brain barrier at a higher rate than semaglutide. -Tested semaglutide and DA5-CH in the 6-OHDA-lesion rat model of PD	-Adult male Sprague-Dawley (SD) rats	-Stereotactic surgery -A sham + saline group -A 6-OHDA+ saline group -A 6-OHDA+ semaglutide group -A 6-OHDA+DA5-CH group -Treatment was semaglutide or DA5-CH (25 nmol/kg, i.p.) daily for 30 days postlesion.	-Both drugs protected dopaminergic neurons and increased TH expression in the substantia nigra. -The level of monomer and aggregated α-synuclein was reduced.
12. X. Yang et al. 2019 [35]	-Providing new insight into the semaglutide neuroprotective effects in the rat brain	-Adult male Sprague –Dawley	-Permanent middle cerebral artery occlusion (PMCAO) model -Sham-operated group -Vehicle control group -Semaglutide group—2 h after MCAO intraperitoneally (10 nmol/kg), -Injected every second day for 1, 7, 14, and 21 days -Tissue sampling after 21 days of observation	-After ischemia, semaglutide treatment improved the functional recovery of rats without affecting blood glucose levels. -The drug reduced inflammation and apoptosis and normalized cell growth signaling and neurogenesis.
13. Poupon- Bejuit et al. 2022 [36]	-Evaluate the therapeutic efficacy of semaglutide in a mouse model of INAD (infantile neuroaxonal dystrophy)	-Pla2g6^−/−^ mice	-In this study, a mouse model with the Pla2g6 knock-in was given semaglutide at three different doses through intraperitoneal injection once a week. The treatment began when the mice were three weeks old, and their survival was monitored and compared to age-matched untreated Pla2g6^−/−^ mice and wild-type mice (WT), which were used as controls.	-Weekly delivery of high-dose semaglutide improved lifespan and locomotor function in juvenile INAD mice. Semaglutide has been shown to increase the levels of molecules that protect the brain while simultaneously decreasing those molecules that promote neurodegeneration. -The expression of mediators in the apoptotic and necroptotic pathways was significantly reduced in mice treated with semaglutide.
14. T.S. Salameh et al. 2020 [37]	-Compare the pharmacokinetics of ^125^I-labeled single IRAs (exendin-4, liraglutide, lixisenatide, semaglutide) and ^125^I-labeled dual IRAs	-Male CD-1 mice	-Consequently, compared brain uptake pharmacokinetics of intravenous ^125^I-labeled IRAs in adult CD-1 mice over 60 min	-The nonacylated and nonpegylated IRAs (exendin-4, lixisenatide, Peptide 17, DA3-CH, and DA-JC4) had significant rates of blood-to-brain influx (Ki). -The acylated IRAs (liraglutide, semaglutide, and Peptide 18) did not measurably cross the BBB. The capillaries completely segregated semaglutide and Peptide 18, and none of the 125I-semaglutide or Peptide 18 was found in the brain tissue.
15. Yan-fang Chang et al. 2020 [41]	-Explore the potential mechanisms of semaglutide against AD	-SH-SY5Y cells	-SH-SY5Y cells damaged by Ab_25–35_ were treated by semaglutide. -Autophagy-related proteins and apoptosis-related proteins were measured to explore molecular mechanisms.	-Semaglutide inhibited apoptosis and increased the expression of Bcl2 inhibited by Ab_25–35_. -Semaglutide activity in preventing SH-SY5Y cells against Ab_25–35_ present a potential mechanism for enhancing autophagy and inhibiting apoptosis.
16. Thornton et al. 2024 [38]	-Identify specific NLRP3-sensitive mechanismscontributing to obesity-induced inflammation -Compare an NLRP3 inhibitor to semaglutide to evaluate its association with systemic inflammatory response, and cerebral gliosis	-Male C57BL/6J mice	-To investigate whether NLRP3 activation contributes to the pathogenesis of diet-induced obesity (DIO) in mice, two different clinical-stage NLRP3 inflammasome inhibitors were tested. -After being on a high-fat diet for 15 weeks, mice induced with diet-induced obesity (DIO) weighed significantly more than the mice fed a regular chow-based diet. To understand the effect of the LRP3 inhibitor NT-0249, it was compared with semaglutide or calorie restriction for an additional 28 days.	-NLRP3 inhibitors can block the cellular inflammatory response to obesity-related molecular patterns. -In pre-existing obese states in mice, inhibiting NLRP3 can reduce systemic inflammation and astrogliosis. When dosed to achieve brain exposure, NLRP3 inhibitors can reverse pre-established obesity at a similar efficacy to the GLP-1RA semaglutide.
17. Shnaien et al. 2023 [39]	-Examine the neuroprotective effects of semaglutide during endotoxemia and its role in modulating pro-inflammatory mediators.	-Adult male Swiss albino mice	-Laparotomy without/with cecal ligation and puncture (CLP) -Semaglutide administration before CLP -Sham group: laparotomy surgery without CLP. -CLP group: (sepsis group) •Vehicle group: mice were given an equal volume of distilled water (DW) by subcutaneous injection Daily for seven days before CLP. •Semaglutide group: mice received 40 µg/kg of semaglutide	-The brain tissue level of TLR4\STAT3 of the semaglutide-treated group was significantly lower. -The brain tissues obtained from mice treated with semaglutide showed significantly less cellular injury. -Semaglutide can protect against sepsis and prevent brain dysfunction.

## Data Availability

All data are available at the corresponding author upon request.

## Supplementary Materials

The following supporting information can be downloaded at https://www.mdpi.com/article/10.3390/ijms25094972/s1.
