# Supplementary material for: A Systematic Review of Semaglutide’s Influence on Cognitive Function in Preclinical Animal Models and Cell-Line Studies
Source: Int J Mol Sci. 2024 May 2;25(9):4972. doi: 10.3390/ijms25094972 (PMC11084700; doi:10.3390/ijms25094972)
Supplement: Supplementary file 1 [file ijms-25-04972-s001.zip › ijms-2941951-supplementary.pdf]

Supplementary File S1

Table S1. SYRCLE's tool for assessing risk of bias [22-24]

	Selection bias	Selection bias	Selection bias	Performance bias	Performance bias	Detection bias	Detection bias	Attrition bias	Reporting bias	Other	
**Study**	**D1 Sequence generation**	**D2 Baseline characteristics**	**D3 Allocation concealment**	**D4 Random housing**	**D5 Blinding**	**D6 Random outcome assessment**	**D7 Blinding**	**D8 Incomplete outcome data**	**D9 Selective outcome reporting**	**D10 Other sources of bias**	**Overall**
Basalay et al. 2019	Low	Low	Not applicable	Low	Unclear	Low	High	Unclear	Unclear	Unclear	Low
Chen et al. 2023a	Low	Low	Not applicable	Low	Unclear	Low	High	Unclear	High	Unclear	Low
Wang et al. 2021	Low	Low	Not applicable	Low	Unclear	Low	High	Unclear	Unclear	Unclear	Low
M.A. Sadek et al. 2023	Low	Low	Not applicable	Low	Unclear	Low	Unclear	Unclear	Unclear	Unclear	Low
Zhang Q. et al. 2022	Low	Low	Not applicable	Low	Unclear	Low	High	Unclear	Unclear	Unclear	Low
Chen et al. 2023b	Low	Low	Not applicable	Low	Unclear	Low	High	Unclear	High	Unclear	Low
Z.J. Wang et al. 2023	Low	Low	Not applicable	Low	Unclear	Low	High	Unclear	High	Unclear	Low
L. Zhang et al. 2018	Low	Low	Not applicable	Low	Unclear	Low	High	Unclear	Unclear	Unclear	Low
L. Zhang et al. 2019	Low	Low	Not applicable	Low	Unclear	Low	High	Unclear	Unclear	Unclear	Low
L. Zhang et al. 2022	Low	Low	Not applicable	Low	Unclear	Low	Unclear	Unclear	High	Unclear	Low
X. Yang et al. 2019	Low	Low	Not applicable	Low	Unclear	Low	High	Unclear	High	Unclear	Low
Poupon-Bejuit et al. 2022	Low	Low	Not applicable	Low	Unclear	Low	High	Unclear	Unclear	Unclear	Low
T.S. Salameh et al.2020	Low	Low	Not applicable	Low	Unclear	Low	Unclear	Unclear	Unclear	Unclear	Low
Thornton et al. 2024	Low	Low	Not applicable	Low	Unclear	Low	Unclear	Unclear	Unclear	Unclear	Low
Shnaien et al. 2023	Low	Low	Not applicable	Low	Unclear	Low	Unclear	Unclear	Unclear	Unclear	Low

D1 Was the allocation sequence adequately generated and applied?

D2 Were the groups similar at baseline or were they adjusted for confounders in the analysis?

D3 Was the allocation adequately concealed?

D4 Were the animals randomly housed during the experiment?

D5 Were the caregivers and /or investigators blinded from knowledge which intervention each animal received during the experiment?

D6 Were animals selected at random for outcome assessment?

D7 Was the outcome assessor blinded?

D8 Were incomplete outcome data adequately addressed?

D9 Are reports of the study free of selective outcome reporting?

D10 Was the study apparently free of other problems that could result in high risk of bias?

Tabel S2. The Risk Of Bias In Non-randomized Studies – of Interventions (ROBINS-I) [22-24]

Study	Bias due to confounding	Bias in selection of participants into the study	Bias in classification of interventions	Bias due to deviations from intended interventions	Bias due to missing data	Bias in measurement of outcomes	Bias in selection of the reported result	Overall
Liu et al. 2022	Moderate	Low	Low	Moderate	Low	Low	Moderate	Low
Yan-fang Char	Moderate	Low	Low	Moderate	Low	Low	Moderate	Low

Supplementary File S2 - PRISMA 2020 Checklist

Section and Topic	Item #	Checklist item	Location where item is reported
**TITLE**			
Title	1	Identify the report as a systematic review.	Page 1
**ABSTRACT**			
Abstract	2	See the PRISMA 2020 for Abstracts checklist.	Page 1; no registration number, protocol is supplied as suppl. material
**INTRODUCTION**			
Rationale	3	Describe the rationale for the review in the context of existing knowledge.	Page 2
Objectives	4	Provide an explicit statement of the objective(s) or question(s) the review addresses.	Page 3
**METHODS**			
Eligibility criteria	5	Specify the inclusion and exclusion criteria for the review and how studies were grouped for the syntheses.	Page 4-5
Information sources	6	Specify all databases, registers, websites, organisations, reference lists and other sources searched or consulted to identify studies. Specify the date when each source was last searched or consulted.	Page 4-5, figure 2
Search strategy	7	Present the full search strategies for all databases, registers and websites, including any filters and limits used.	Page 4-5, figure 2
Selection process	8	Specify the methods used to decide whether a study met the inclusion criteria of the review, including how many reviewers screened each record and each report retrieved, whether they worked independently, and if applicable, details of automation tools used in the process.	Page 4-5
Data collection process	9	Specify the methods used to collect data from reports, including how many reviewers collected data from each report, whether they worked independently, any processes for obtaining or confirming data from study investigators, and if applicable, details of automation tools used in the process.	Page 4
Data items	10a	List and define all outcomes for which data were sought. Specify whether all results that were compatible with each outcome domain in each study were sought (e.g. for all measures, time points, analyses), and if not, the methods used to decide which results to collect.	Page 4-5, figure 2
	10b	List and define all other variables for which data were sought (e.g. participant and intervention characteristics, funding sources). Describe any assumptions made about any missing or unclear information.	Page 5, figure 2
Study risk of bias assessment	11	Specify the methods used to assess risk of bias in the included studies, including details of the tool(s) used, how many reviewers assessed each study and whether they worked independently, and if applicable, details of automation tools used in the process.	Page 5, figure 3, figure 4
Effect measures	12	Specify for each outcome the effect measure(s) (e.g. risk ratio, mean difference) used in the synthesis or presentation of results.	Table 1 (page 7-10)
Synthesis methods	13a	Describe the processes used to decide which studies were eligible for each synthesis (e.g. tabulating the study intervention characteristics and comparing against the planned groups for each synthesis (item #5)).	Page 4-5
	13b	Describe any methods required to prepare the data for presentation or synthesis, such as handling of missing summary statistics, or data conversions.	Figure 2

Section and Topic	Item #	Checklist item	Location where item is reported
	13c	Describe any methods used to tabulate or visually display results of individual studies and syntheses.	Table 1
	13d	Describe any methods used to synthesize results and provide a rationale for the choice(s). If meta-analysis was performed, describe the model(s), method(s) to identify the presence and extent of statistical heterogeneity, and software package(s) used.	Table 1. no meta-analysis performed
	13e	Describe any methods used to explore possible causes of heterogeneity among study results (e.g. subgroup analysis, meta-regression).	Page 6, table 1
	13f	Describe any sensitivity analyses conducted to assess robustness of the synthesized results.	Figure 2
Reporting bias assessment	14	Describe any methods used to assess risk of bias due to missing results in a synthesis (arising from reporting biases).	Supplementary File 1, figure 3, figure 4
Certainty assessment	15	Describe any methods used to assess certainty (or confidence) in the body of evidence for an outcome.	Supplementary File 1, figure 3, figure 4
**RESULTS**			
Study selection	16a	Describe the results of the search and selection process, from the number of records identified in the search to the number of studies included in the review, ideally using a flow diagram.	Figure 2
	16b	Cite studies that might appear to meet the inclusion criteria, but which were excluded, and explain why they were excluded.	Page 6-7
Study characteristics	17	Cite each included study and present its characteristics.	Table 1
Risk of bias in studies	18	Present assessments of risk of bias for each included study.	Figure 3 and 4
Results of individual studies	19	For all outcomes, present, for each study: (a) summary statistics for each group (where appropriate) and (b) an effect estimate and its precision (e.g. confidence/credible interval), ideally using structured tables or plots.	Table 1
Results of syntheses	20a	For each synthesis, briefly summarise the characteristics and risk of bias among contributing studies.	Supplementary File 1
	20b	Present results of all statistical syntheses conducted. If meta-analysis was done, present for each the summary estimate and its precision (e.g. confidence/credible interval) and measures of statistical heterogeneity. If comparing groups, describe the direction of the effect.	No statistical synthesis conducted.
	20c	Present results of all investigations of possible causes of heterogeneity among study results.	Table 1
	20d	Present results of all sensitivity analyses conducted to assess the robustness of the synthesized results.	Page 11-16
Reporting biases	21	Present assessments of risk of bias due to missing results (arising from reporting biases) for each synthesis assessed.	Figure 3
Certainty of evidence	22	Present assessments of certainty (or confidence) in the body of evidence for each outcome assessed.	Page 11-16
**DISCUSSION**			
Discussion	23a	Provide a general interpretation of the results in the context of other evidence.	Page 11-16
	23b	Discuss any limitations of the evidence included in the review.	Page 17
	23c	Discuss any limitations of the review processes used.	Page 17
	23d	Discuss implications of the results for practice, policy, and future research.	Page 16-17
**OTHER INFORMATION**			

Section and Topic	Item #	Checklist item	Location where item is reported
Registration and protocol	24a	Provide registration information for the review, including register name and registration number, or state that the review was not registered.	the review was not registered
	24b	Indicate where the review protocol can be accessed, or state that a protocol was not prepared.	the review protocol cannot be accessed
	24c	Describe and explain any amendments to information provided at registration or in the protocol.	Page 17
Support	25	Describe sources of financial or non-financial support for the review, and the role of the funders or sponsors in the review.	Page 17
Competing interests	26	Declare any competing interests of review authors.	Page 17
Availability of data, code and other materials	27	Report which of the following are publicly available and where they can be found: template data collection forms; data extracted from included studies; data used for all analyses; analytic code; any other materials used in the review.	Page 17

From: Page MJ, McKenzie JE, Bossuyt PM, Boutron I, Hoffmann TC, Mulrow CD, et al. The PRISMA 2020 statement: an updated guideline for reporting systematic reviews. BMJ 2021;372:n71. doi: 10.1136/bmj.n71

Supplementary File S3 - PRISMA 2020 for Abstracts Checklist

Section and Topic	Item #	Checklist item	Reported (Yes/No)
**TITLE**			
Title	1	Identify the report as a systematic review.	Yes
**BACKGROUND**			
Objectives	2	Provide an explicit statement of the main objective(s) or question(s) the review addresses.	Yes
**METHODS**			
Eligibility criteria	3	Specify the inclusion and exclusion criteria for the review.	No
Information sources	4	Specify the information sources (e.g. databases, registers) used to identify studies and the date when each was last searched.	Yes
Risk of bias	5	Specify the methods used to assess risk of bias in the included studies.	Yes
Synthesis of results	6	Specify the methods used to present and synthesise results.	No
**RESULTS**			
Included studies	7	Give the total number of included studies and participants and summarise relevant characteristics of studies.	Yes
Synthesis of results	8	Present results for main outcomes, preferably indicating the number of included studies and participants for each. If meta-analysis was done, report the summary estimate and confidence/credible interval. If comparing groups, indicate the direction of the effect (i.e. which group is favoured).	Yes
**DISCUSSION**			
Limitations of evidence	9	Provide a brief summary of the limitations of the evidence included in the review (e.g. study risk of bias, inconsistency and imprecision).	Yes
Interpretation	10	Provide a general interpretation of the results and important implications.	Yes
**OTHER**			
Funding	11	Specify the primary source of funding for the review.	No
Registration	12	Provide the register name and registration number.	No

From: Page MJ, McKenzie JE, Bossuyt PM, Boutron I, Hoffmann TC, Mulrow CD, et al. The PRISMA 2020 statement: an updated guideline for reporting systematic reviews. BMJ 2021;372:n71. doi: 10.1136/bmj.n71
